# Altered patterns in the prefrontal cortex and limbic system in female patients with neuropathic pain secondary to recurrent median nerve entrapment post carpal tunnel release

**DOI:** 10.1093/braincomms/fcaf375

**Published:** 2025-09-26

**Authors:** Beibei Feng, Chen Gong, William Weijia Lu, Yuling Wang, Wing-Yuk Ip

**Affiliations:** Department of Rehabilitation Medicine, Sixth Affiliated Hospital, Sun Yat-sen University, Guangzhou 510655, China; Department of Orthopaedics & Traumatology, The University of Hong Kong, 999077 Hong Kong SAR, China; Guangdong Provincial Clinical Research Center for Rehabilitation Medicine, Guangzhou 510655, China; Biomedical Innovation Center, the Sixth Affiliated Hospital, Sun Yat-sen University, Guangzhou 510655, China; Department of Rehabilitation Medicine, Sixth Affiliated Hospital, Sun Yat-sen University, Guangzhou 510655, China; Guangdong Provincial Clinical Research Center for Rehabilitation Medicine, Guangzhou 510655, China; Biomedical Innovation Center, the Sixth Affiliated Hospital, Sun Yat-sen University, Guangzhou 510655, China; Department of Orthopaedics & Traumatology, The University of Hong Kong, 999077 Hong Kong SAR, China; Department of Rehabilitation Medicine, Sixth Affiliated Hospital, Sun Yat-sen University, Guangzhou 510655, China; Guangdong Provincial Clinical Research Center for Rehabilitation Medicine, Guangzhou 510655, China; Biomedical Innovation Center, the Sixth Affiliated Hospital, Sun Yat-sen University, Guangzhou 510655, China; Department of Orthopaedics & Traumatology, The University of Hong Kong, 999077 Hong Kong SAR, China

**Keywords:** carpal tunnel syndrome, recurrent neuropathic pain, functional magnetic resonance imaging, prefrontal cortex, limbic system

## Abstract

Abnormal central pain sensitization is a possible explanation for persistent neuropathic pain after carpal release. However, its exact mechanism remains unclear. This study examined the cortical patterns in chronic neuropathic pain secondary to recurring median neuropathy. Forty-nine patients with recurrent painful carpal tunnel syndrome and 22 age- and sex-matched controls were enrolled. Multimodal MRI, including structural, blood oxygenation level-dependent, and three-dimensional pseudo-continuous arterial spin labelling were employed. Cerebral blood flow and seed-based functional connectivity were analysed. Global and regional cerebral blood flow in several brain regions in the recurring carpal tunnel syndrome group decreased. Significant between-group differences were found in cerebral blood flow maps involving the frontal gyrus and insula, which exhibited an inverse correlation with central sensitization scores. Increased brain connectivity was noted in recurrent carpal tunnel syndrome patients and was negatively correlated with symptom severity. Individuals with chronic neuropathic pain secondary to recurrent median nerve entrapment manifested maladaptive brain networks in cortical regions involving sensory, cognitive and affective processing. Our findings may highlight new avenues for cortical biomarkers and potential mechanisms underlying central sensitization in persistent pain after peripheral neuropathy, which may facilitate innovative targeted management strategies for those who respond poorly to concurrent treatment regimes.

## Teaser

Feng *et al*. report that individuals with chronic neuropathic pain secondary to recurrent carpal tunnel syndrome show decreased cerebral blood flow and altered brain connectivity in the prefrontal cortex and limbic system, which correlates with pain severity. This study provides new insights for centrally mediating strategies for optimal neuropathic pain management.

## Graphical Abstract

**Graphical Abstract:**
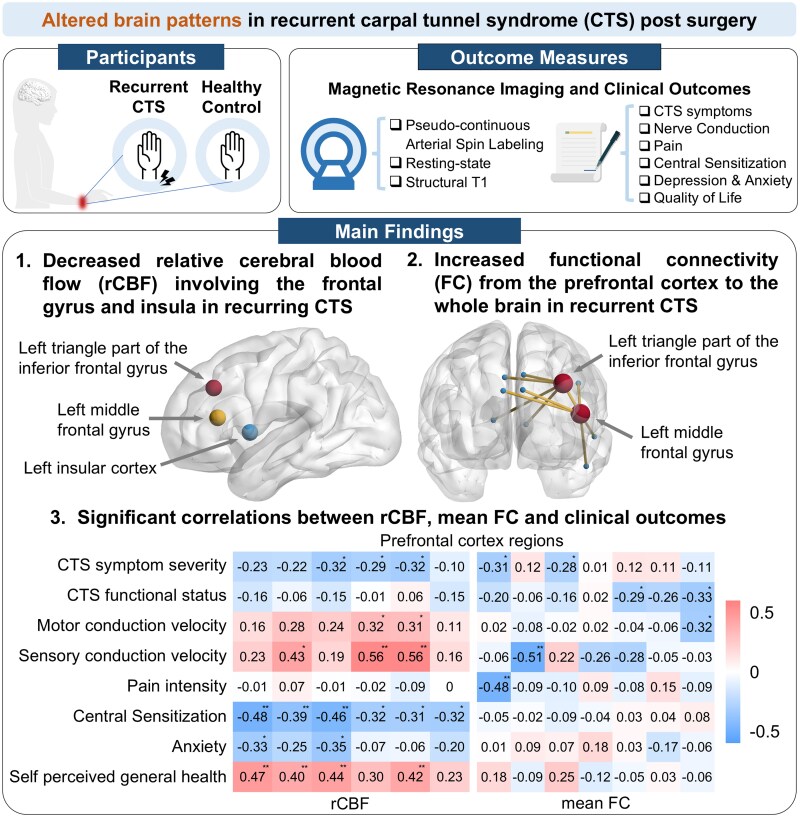

## Introduction

Caused by median nerve compression, carpal tunnel syndrome (CTS) is one of the most prevalent peripheral nerve entrapment disorders.^1,2^ CTS is characterized by ongoing symptoms of numbness, tingling and pain in areas innervated by the median nerve, mostly occurring nocturnally, as well as thenar muscle wasting.^2,3^ Those with moderate or severe median nerve entrapment or who respond poorly to non-surgical interventions or who suffer from functional limitations at work or during daily activities usually require carpal tunnel release surgery, which helps release the intra-tunnel pressure and alleviate the symptoms.^2,4^ However, quite a number of patients still suffer from chronic neuropathic pain even after release procedures.^5^ Recurrent CTS symptoms have been frequently reported after carpal tunnel release surgeries.^6,7^ Patients with persistent neuropathic pain secondary to recurrent median nerve entrapment are usually associated with comorbid anxiety and/or depression, resulting in unsatisfactory prognoses and undermined health-related quality of life.^2,8^ The refractory neuropathic pain in peripheral nerve impairment may be due to abnormal pain sensitization in the central nervous system.^5,9-11^

Understanding the central neuroplasticity in those with recurring neuropathic pain may help elucidate the predictors related to the recurrence of CTS after surgery. Nevertheless, the exact cortical plasticity patterns underlying persistent neuropathic pain secondary to recurrent median nerve entrapment remain not clear hitherto. The brain alterations in primary CTS have been revealed by previous relevant studies. Previous researchers employed task-evoked fMRI to focus on the hand movement-related brain activation in the region of primary somatosensory cortex (S1) and found altered structural and functional plasticity, especially in S1.^12-14^ Changes in brain connectivity were also detected in the S1/primary motor cortex (M1) to whole-brain functional connectivity (FC), as well as abnormal structural connectivity and structural covariance networks among those with CTS.^10,15-18^ However, previous studies merely focused on the general cortical profile of the CTS population. Little is known about those with refractory neuropathic pain secondary to recurring median nerve impairment.

Therefore, the present study aimed to employ a multimodal magnetic resonance imaging (MRI) approach to investigate cortical mediating patterns in chronic neuropathic pain patients subject to recurrent median nerve entrapment. The associations between the central patterns and clinical outcomes in those with painful recurrent CTS were evaluated.

## Materials and methods

### Study design

This was a prospective, observational study with consecutive convenience sampling of CTS patients in two public hospitals in Hong Kong from December 2020 to May 2023. The first patient was enrolled on 28 December 2020. Written consent was obtained from all participants prior to their engagement in the study. All procedures were conducted in accordance with the Declaration of Helsinki.

Research ethics approval was obtained from the Institutional Review Board of the University of Hong Kong/Hospital Authority Hong Kong West Cluster (Ref. No. UW 20-297), and this study was preregistered in the Clinical Trials Registry of The University of Hong Kong (Ref. No. HKUCTR-2828; date of registration, 24 April 2020; URL, https://www.hkuctr.com/Study/Show/286af752b1f54c5ea6fdc84a94759368).

### Sampling

Totally, 75 participants were recruited for the study, including 50 patients with recurrent CTS and 25 healthy controls (HCs). All the participants were right-hand dominant, which eliminated the effect of handedness on the lateralization of brain plasticity. All the CTS patients were bilaterally affected, and the majority presented more severe symptoms in the right hand.

The inclusion criteria for participants with recurring painful CTS were as follows: (i) clinically diagnosed with CTS confirmed by a nerve conduction study (NCS) (median nerve distal motor latency > 4.5 ms and/or median nerve sensory or motor conduction velocity < 50 m/s) and positive provocative tests (Phalen’s test and Tinel sign); (ii) the duration of pain lasting no less than 6 months; (iii) the pain severity score greater than 4 out of 10 on a visual analogue scale (VAS); and (iv) having a history of carpal release surgery at least 1 year prior. The confirmatory diagnosis of recurrent CTS in the present study was performed by an experienced physician in the neurophysiological laboratory, taking into account of mainly three dimensions, namely, the clinical (prior history of carpal tunnel release, a symptom-free/relieving interval and reappearance of typical CTS symptoms and/or positive physical exams such as Tinel’s sign or Phalen’s test), electrophysiological (nerve conduction studies) and optional ultrasound imaging where appropriate.

Those with recurrent CTS meeting any of the following exclusion criteria were excluded: (i) history of wrist or hand surgeries or trauma within the past 6 months or intra-articular injection of corticosteroids within the past 3 months; (ii) comorbidities, including gout, diabetes mellitus, ischaemic heart disease, hypertension, chronic kidney disease, cancer, cervical radiculopathy, myelopathy, neurological diseases or other chronic pain disorders; (iii) abnormal mental status or cognitive problems, unable to comply with the study protocols; (iv) bleeding disorders; and (v) MRI contraindications, such as dentures or cardiac pacemakers.

In addition, a group of age- and sex-matched pain-free HCs was enrolled. The participants in the HC group were right-handed, healthy individuals who had no pain complaints in the past 6 months. The exclusion criteria for the HC group were similar to those for the recurrent CTS group.

In terms of the sample size estimate, given a liberal threshold of 0.05, around 12 individuals were needed for a statistical power of 0.8 at the single-voxel level for typical brain activations, and the sample size may increase accordingly if multiple comparisons are performed.^19^  *A priori* sample size estimate was calculated assuming alpha = 0.05, power = 80% and a moderate effect size prior to recruitment using G*Power [University of California, Los Angeles (UCLA): Statistical Consulting Group, from https://stats.oarc.ucla.edu/other/gpower]. Four subjects were excluded due to image artefacts or low quality, and finally 71 participants, including 49 with CTS and 22 HCs, were included in the analysis. With the current sample size, we have calculated the *post hoc* statistical power of this study, which is 95%.

### Clinical outcome measures

Eligible participants enrolled were asked to complete a series of clinical assessments, including pain severity by visual analogue scale (VAS),^20^ CTS-specific symptomatology and functional ability through the Boston Carpal Tunnel Syndrome Questionnaire (BCTQ)^2,3^ and pain sensitization via the Central Sensitization Inventory (CSI).^21^ The mental status and self-perceived general health were evaluated by the Hospital Anxiety and Depression Scale (HADS)^22^ and EuroQol-5D (EQ-5D),^23^ respectively.

The participants with CTS symptoms were required to undergo a standardized NCS to confirm the clinical degree of severity of both the sensory and motor components of the median nerve via an EMG workstation (Nicolet Viking EDX EMG/EP/NCS system). The method/protocol for the nerve conduction study was performed as previously published.^24,25^

The severity grading of CTS, ranging from mild to severe, was performed by an experienced physician depending on the NCV results, including the distal motor latency of the median motor nerve and/or the conduction velocity and amplitude of the median sensory nerve.^26,27^

### MRI data acquisition

MRI scanning was conducted with a 3.0 T GE MRI scanner (SIGNA Premier, GE HealthCare, USA) with a 48-channel head coil. All the participants were asked to lie down and relax during the scanning process. During the resting-state fMRI scan, they were instructed to rest with their eyes open, look at the cross at the top, not to think of anything in particular and to be awake. Earplugs were given to the participants to reduce noise, and foam pads were provided to maintain a stable head position to minimize head motion.

A three-dimensional pseudo-continuous ASL (pCASL) sequence with a fast spin-echo acquisition and background suppression was applied for the perfusion imaging: repetition time (TR) = 4,932 ms; echo time (TE) = 52.8 ms; flip angle (FA) = 111°; field of view (FOV) = 240 × 240 mm^2^; post-label delay (PLD) = 2025 ms; reconstruction matrix = 240 × 240; slice thickness = 4 mm; and spacing between slices = 4. Forty-two pairs of labelled and control images were collected for the calculation of the CBF.

The resting-state fMRI images were obtained using an echo-planar imaging (EPI) pulse sequence: TR = 2500 ms; TE = 30 ms; FA = 90°; slice number = 49; slice thickness = 3 mm; spacing between slices = 3; FOV = 288 × 288 mm^2^; matrix size = 128 × 128; timepoints = 204; number of acquisitions = 9996; and scanning duration = 8.5 min.

Moreover, structural 3D SAG T1 MPRAGE images were acquired via the BRAVO sequence: TR = 7 ms; TE = 3 ms; inversion time = 900 ms; FA = 80; matrix size = 512 × 512; FOV = 240 × 240 mm^2^; slice thickness = 1.00 mm; spacing between slices = 0.5; number of acquisitions = 332; and number of excitations = 1.0. Structural T1 scanning was used to screen and exclude those with any anatomical brain abnormalities. No participants enrolled were found with any structural dysfunctions.

### Data processing

All MRI image processing and analyses were performed on the MATLAB platform (MATLAB version 2021b, MathWorks Inc., Natick, MA, USA).

The pCASL perfusion images were preprocessed via a modified processing pipeline, ASLtbx,^28^ which is based on the Statistical Parametric Mapping 12 (SPM12, Functional Imaging Laboratory, Oxford, UK). The preprocessing for pCASL MRI images included the following steps: (i) reorientation for all images, including structural T1 images, M0 images and CBF images; (ii) motion correction, wherein rigid body transformation was adopted to estimate the motion time courses for all control and label images, and the zigzag pattern was then regressed out; (iii) coregistration between pCASL images and structural T1 images, where the pCASL images were coregistered to anatomical images before they were spatially normalized to the Montreal Neurological Institute (MNI) space; and (iv) smoothing, wherein the normalized CBF maps were smoothed by using a 6 mm full width at half maximum (FWHM) Gaussian kernel. After preprocessing, a mask based on the mean of the functional ASL images was generated, which was used to exclude those out-of-brain voxels. Finally, CBF quantification was performed to calculate the global mean CBF, relative CBF (relCBF) and regional CBF (rCBF) in different brain regions using the AAL template.^29^

Resting-state fMRI data were preprocessed via Data Processing and Analysis for (Resting-State) Brain Imaging (DPABI) based on SPM12.^30^ The initial 10 time points were discarded for signal equilibrium, and slice timing was applied to correct different signal acquisition times for all volume slices. Then, realignments were performed via a six-parameter rigid body transformation, followed by the process of co-registering T1 images to the mean functional images using a 6-degree-of-freedom linear transformation. Then, the brain images were segmented into grey matter, white matter and cerebrospinal fluid. Images were spatially registered from individual native space to the MNI space using the Diffeomorphic Anatomical Registration Through Exponentiated Lie (DARTEL) algebra computation. Finally, spatial smoothing was conducted for all functional images with a 6 mm FWHM Gaussian kernel. The nuisance signals from the white matter and cerebrospinal fluid, as well as the Friston 24 head motion parameters, were regressed out to minimize the confounding effects. Band-pass filtering (0.01–0.08 Hz) and linear detrending were performed to control the low-frequency drifts and high-frequency artefacts. Any participant whose average frame displacement was greater than 0.2 mm in Jenkinson *et al*.’s calculation approach was excluded. In this study, 4 participants, including one CTS patient and 3 HCs, were excluded because of artefacts or excessive head movement.

Seed-based functional connectivity was conducted using the DPABI toolbox.^30^ Seed regions were selected in accordance with previous relevant studies concerning persistent neuropathic pain, which focused mainly on the prefrontal cortex (PFC).^31-34^ Correlational analyses were conducted to evaluate the associations between cortical changes, including brain perfusion and functional connectivity, and clinical outcomes in patients with recurring CTS.

### Statistical analysis

Demographics, clinical outcome parameters, global and regional CBF values and the mean FC extracted were compared between participants with recurrent CTS and HCs. The data distribution and normality in the study were tested using the Shapiro–Wilk’s test before further statistical analyses. Descriptive statistics were employed to present the demographic and clinical characteristics of the participants. Two-sample *t*-test or Mann–Whitney *U* test was used to conduct groupwise comparisons depending on the nature and normality of the variables for parametric or non-parametric statistics. The statistical analyses of the clinical data were performed using version 27.0 of the IBM® SPSS® Statistics software (IBM Corp., NY, USA). The significance level was set at 0.05.

Furthermore, a voxel-wise comparison of CBF brain maps was conducted between the CTS and HC groups via a two-sample *t*-test, with age controlled. In terms of the FC maps, a one-sample *t*-test was adopted for each seed region in either group for within-group FC spatial distribution. The between-group differences were compared within the mask defined as the aggregated mask of one-sample *t*-test maps of the two groups. To minimize multiple comparison errors, all the statistical results of the two-sample *t*-test were corrected by Gaussian random field (GRF) theory, with voxel *P* < 0.001, cluster *P* < 0.05, two-tailed.

ROI-based FC analyses were performed based on the preset target regions related to chronic neuropathic pain.^31-34^ The correlations between significant neuronal activation-related CBF changes (relCBF and rCBF) as well as whole-brain connectivity and the clinical outcomes of those with CTS were then investigated using Pearson or Spearman correlation analyses depending on the data distribution and normality. All correlational analyses employed partial correlation controlling for the variable of age, and the multiple comparisons were corrected by GRF with voxel *P* < 0.001, cluster *P* < 0.05, two-tailed.

## Results

### Demographic and clinical characteristics of the participants

Totally 71 participants, with 49 recurrent CTS patients and 22 HCs, were included in the data analysis. In the recurring CTS group, all participants had bilateral CTS, with the right side being more affected, and the average symptom duration was 36.4 months. The majority of patients were classified as moderate, moderate to severe or severe in terms of the clinical severity of CTS confirmed by NCS. The average pain intensity was 6.3 out of 10 on VAS in patients with CTS. The average time since the last carpal tunnel release surgery in the recurring CTS group was 2.26 years.

There was no significant difference between recurrent CTS patients and the controls in either age or sex. Significant between-group differences were found in terms of body mass index (BMI), CSI total score, anxiety and depression score and self-perceived general health.

The demographic and clinical characteristics of the participants are summarized in Table 1.

**Table 1:** Demographic and clinical characteristics of the participants

	CTS group (*n* = 49)	HC group (*n* = 22)	*P* value
Age (years, mean (SD))	59.8 (11.22)	57.7 (4.90)	0.246
Sex (female, *n*)	49	22	NA
BMI (mean (SD))	25.3 (4.43)	21.4 (2.35)	<0.001*
Symptom duration [month, mean (SD)]	36.4 (33.50)	NA	NA
Time since last CTR surgery [year, mean (SD)]	2.3 (1.23)	NA	NA
CTS severity level, *n* (%)			
Severe	29 (59.2)	NA	NA
Moderate to severe	10 (20.4)	NA	NA
Moderate	8 (16.3)	NA	NA
Mild to moderate	2 (4.1)	NA	NA
Mild	0	NA	NA
BCTQ scores [mean (SD)]			
Symptom severity score	30.3 (7.33)	NA	NA
Function status score	17.9 (4.84)	NA	NA
Average pain intensity in the past month	6.3 (1.31)	NA	NA
Current pain intensity	5.7 (1.87)	NA	NA
Maximal pain intensity in the past month	8.0 (1.38)	NA	NA
CSI total score [mean (SD)]	36.1 (11.28)	17.3 (12.12)	<0.001*
Anxiety score [mean (SD)]	6.1 (4.10)	2.5 (2.84)	<0.001*
Depression score [mean (SD)]	6.2 (4.59)	2.0 (2.23)	<0.001*
Self-perceived general health [mean (SD)]	60.3 (13.17)	83.6 (14.65)	<0.001*

Abbreviations: CTR, carpal tunnel release; CTS, carpal tunnel syndrome; HC, healthy control; SD, standard deviation; BMI, body mass index; BCTQ, Boston Carpal Tunnel Questionnaire; CSI, central sensitization inventory.

^*^represents *P* < 0.05 for between-group comparisons.

### Global CBF between groups

The global CBF in recurring CTS patients was significantly lower than that in HCs (*P* = 0.024) (Supplemental Table S1). In the voxel-wise analysis of the global mean CBF map, the significance in the two-sample *t*-test did not persist after multiple comparison correction by GRF (voxel *P* < 0.001, cluster *P* < 0.05), taking age as the covariate.

### relCBF map between groups

**Figure 1:**
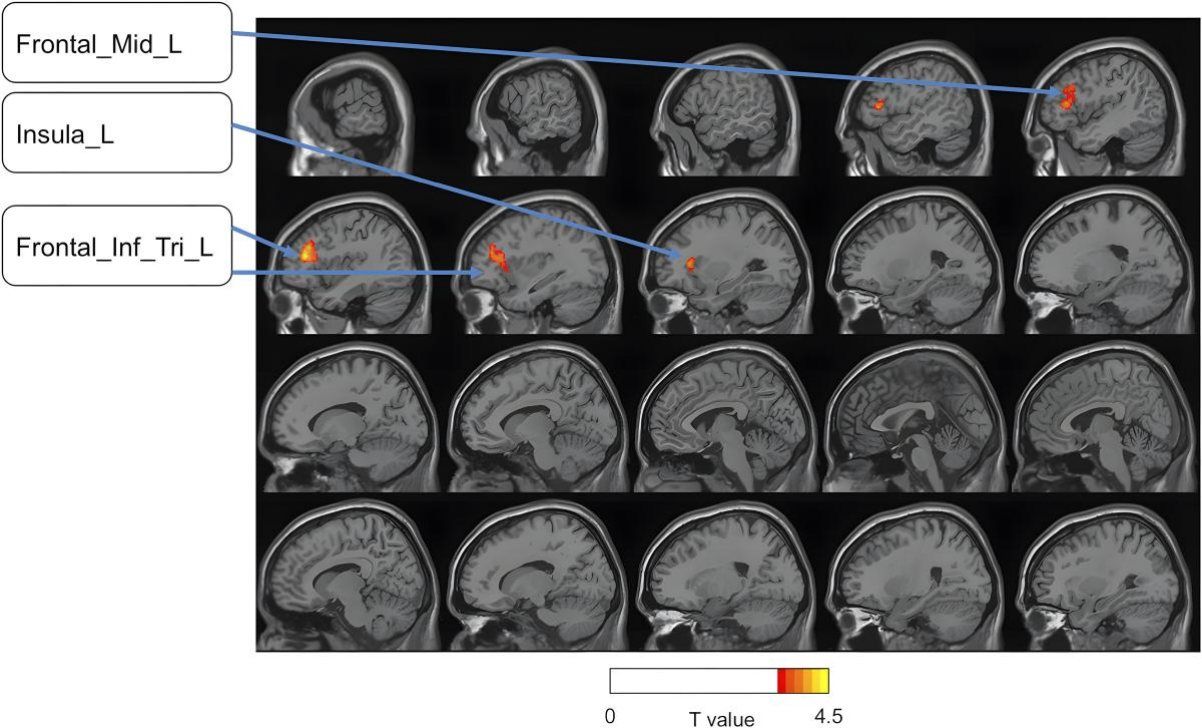
**RelCBF map differences between CTS patients and HCs (sagittal view).** The performed statistical test is a *t*-test with *T*-values ranging from 1 to 4.5 between healthy controls (*n* = 22) and recurrent CTS patients (n = 49). Abbreviations: Frontal_Inf_Tri_L, left triangle part of the inferior frontal gyrus; Frontal_Mid_L, left middle frontal gyrus; Insula_L, left insular cortex.

Since the absolute global CBF may be confounded by different factors, such as nuisance signals or other individual factors, relCBF maps, in relation to the global mean CBF, are commonly adopted as a more reliable parameter to measure cerebral perfusion conditions.^35^ In our study, significant differences were found in the relCBF between the two groups, using a two-sample *t*-test with age as the covariate and multiple comparisons corrected by the GRF (voxel *P* < 0.001, cluster *P* < 0.05, two-tailed). The brain regions showing significantly different relCBFs between recurring CTS patients and controls included the left (contralateral) inferior and middle frontal gyrus and the insular cortex. Figure 1 displays brain areas with altered relCBF maps across the two groups. Details with MNI coordinates are presented in Supplemental Table S2.

### Regional CBF between groups

Table 2 presents the rCBF values in different brain regions of interest between recurrent CTS patients and HCs. Significant between-group differences in rCBF were observed in brain areas, including the left (contralateral) inferior frontal gyrus, left middle frontal gyrus and left superior frontal gyrus, as well as the left parahippocampus and thalamus.

**Table 2:** Regional CBF in different brain regions of interest between the CTS and HC groups

Brain regions	Hemisphere	CTS, mean (SD)	HC, mean (SD)	*P* value
Amygdala	Left	40.2 (6.78)	43.3 (5.17)	0.060
	Right	36.9 (5.56)	36.5 (5.69)	0.786
Frontal_Inf_Oper	Left	50.3 (8.46)	55.0 (7.96)	0.031*
	Right	40.4 (6.41)	41.2 (6.61)	0.652
Frontal_Inf_Orb	Left	49.5 (7.87)	54.6 (6.20)	0.008*
	Right	39.5 (6.30)	39.9 (5.06)	0.796
Frontal_Inf_Tri	Left	49.1 (8.92)	54.0 (7.36)	0.029*
	Right	33.7 (5.55)	33.5 (4.96)	0.866
Frontal_Mid	Left	43.8 (8.64)	47.1 (6.86)	0.127
	Right	33.9 (6.36)	34.2 (5.89)	0.840
Frontal_Sup	Left	33.6 (6.83)	36.4 (5.44)	0.095
	Right	31.4 (5.95)	31.7 (4.88)	0.825
Frontal_Mid_Orb	Left	48.0 (9.10)	52.9 (7.18)	0.027*
	Right	44.4 (8.26)	48.2 (5.87)	0.055
Frontal_Sup_Orb	Left	41.3 (8.43)	45.4 (5.68)	0.039*
	Right	38.8 (7.78)	41.1 (5.52)	0.213
Frontal_Sup_Medial	Left	36.3 (7.54)	38.8 (5.76)	0,174
	Right	34.4 (6.71)	36.1 (4.11)	0.256
Hippocampus	Left	43.6 (7.21)	47.0 (6.22)	0.066
	Right	38.9 (5.11)	40.5 (5.58)	0.249
Parahippocampus	Left	48.6 (7.81)	53.4 (6.94)	0.017*
	Right	46.8 (7.39)	50.2 (7.48)	0.079
Insula	Left	45.2 (7.40)	48.1 (6.91)	0.121
	Right	47.3 (6.25)	48.3 (6.86)	0.576
Postcentral	Left	42.8 (8.09)	45.6 (7.57)	0.180
	Right	34.5 (6.54)	35.5 (5.92)	0.575
Precentral	Left	42.2 (7.87)	45.1 (6.45)	0.126
	Right	35.4 (6.59)	35.6 (5.90)	0.940
Thalamus	Left	44.4 (9.19)	48.9 (7.17)	0.047*
	Right	42.7 (8.39)	45.4 (5.40)	0.113

Abbreviations: CBF, cerebral blood flow; CTS, carpal tunnel syndrome; HC, healthy control; SD, standard deviation; Lt, left; Rt, right; Frontal_Inf_Oper, inferior frontal gyrus, opercular part; Frontal_Inf_Orb, inferior frontal gyrus, orbital part; Frontal_Inf_Tri, inferior frontal gyrus, triangular part; Frontal_Mid, middle frontal gyrus; Frontal_Sup, superior frontal gyrus; Frontal_Mid_Orb, middle frontal gyrus, orbital part; Frontal_Sup_Orb, superior frontal gyrus, orbital part; Frontal_Sup_Med, superior frontal gyrus, medial.

^*^represents *P* < 0.05 for between-group comparisons.

### Seed-based functional connectivity between groups

Voxel-wise seed-based FC analyses were performed with the preset target seed regions of the PFC as indicated in previous studies related to chronic neuropathic pain as the seed regions.^31-34^ In the present study, similarly, we found two parts of the PFC regions were of statistical differences in the relCBF between the two groups, namely the left inferior frontal gyrus and left middle frontal gyrus, which were selected as seeds. Significantly increased FC was found in both the left inferior frontal gyrus and the left middle frontal gyrus to whole-brain functional connectivity in recurrent CTS patients, compared with the controls. The comparisons were examined by a two-sample *t*-test with age adjusted and multiple comparisons corrected by the GRF (voxel *P* < 0.001, cluster *P* < 0.05, two-tailed). What is more, given the important role of the anterior cingulate cortex (ACC) and insula areas in the limbic system, seed-based FC analyses were also conducted with ACC and insula as the seed regions. However, no significant between-group differences were observed in the above functional connectivity.

**Figure 2:**
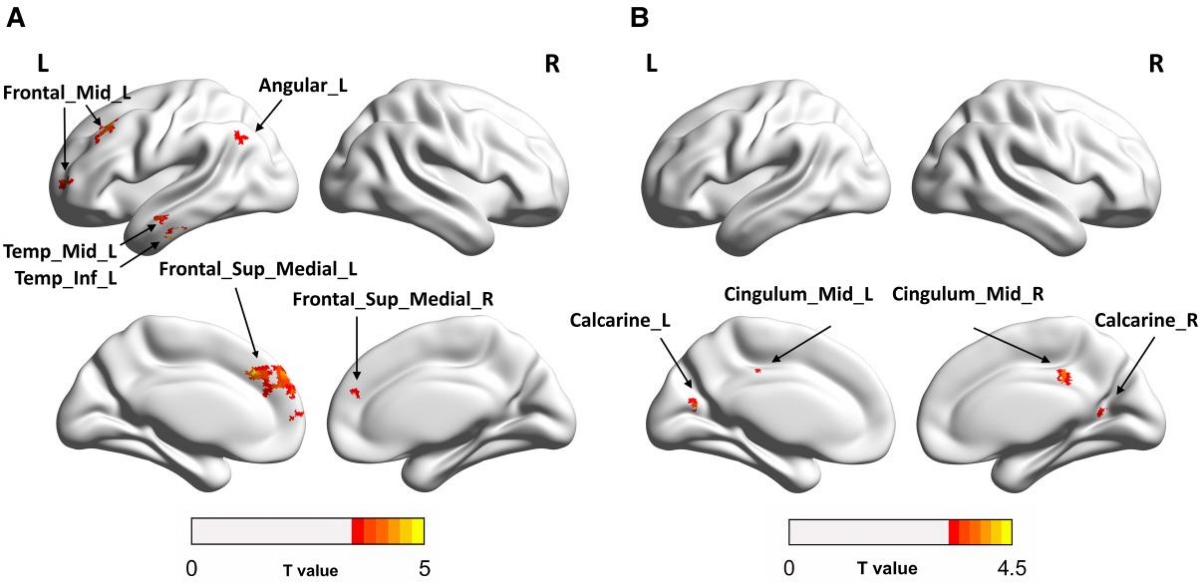
**Functional connectivity differences between CTS patients and HCs from the Frontal_Inf_Tri_L and Frontal_Mid_L to the whole brain.** (**A**) Brain regions of significant whole-brain FC between groups with Frontal_Inf_Tri_L as the seed. (**B**) Brain regions of significant whole-brain FC between groups with Frontal_Mid_L as the seed. Group differences were examined using the *t*-test with *T*-values ranging from 1 to 4.5 or 1 to 5 between healthy controls (*n* = 22) and recurrent CTS patients (*n* = 49). Abbreviations: Angular_L, left angular gyrus; Calcarine_L, left calcarine fissure and surrounding cortex; Calcarine_R, right calcarine fissure and surrounding cortex; Cingulum_Mid_L, left middle cingulate and paracingulate gyri; Cingulum_Mid_R, right middle cingulate and paracingulate gyri; Frontal_Mid_L, left middle frontal gyrus; Frontal_Sup_Medial_L, left superior frontal gyrus, medial part; Frontal_Sup_Medial_R, right superior frontal gyrus, medial part; Temp_Mid_L, left middle temporal gyrus; Temp_Inf_L, left inferior temporal gyrus.

Figure 2 shows the brain regions with altered FC maps between the two groups. The clusters of significance in FC are summarized in Supplemental Tables S3–S4.

### Correlational analyses

Negative associations were found between the relCBF map and clinical parameters, including the central sensitization score by CSI (Supplemental Table S5), anxiety and depression score by HADS in those with CTS (Supplemental Table S6–S7), while a positive correlation was seen between relCBF and self-perceived general health (Supplemental Table S8).

**Figure 3:**
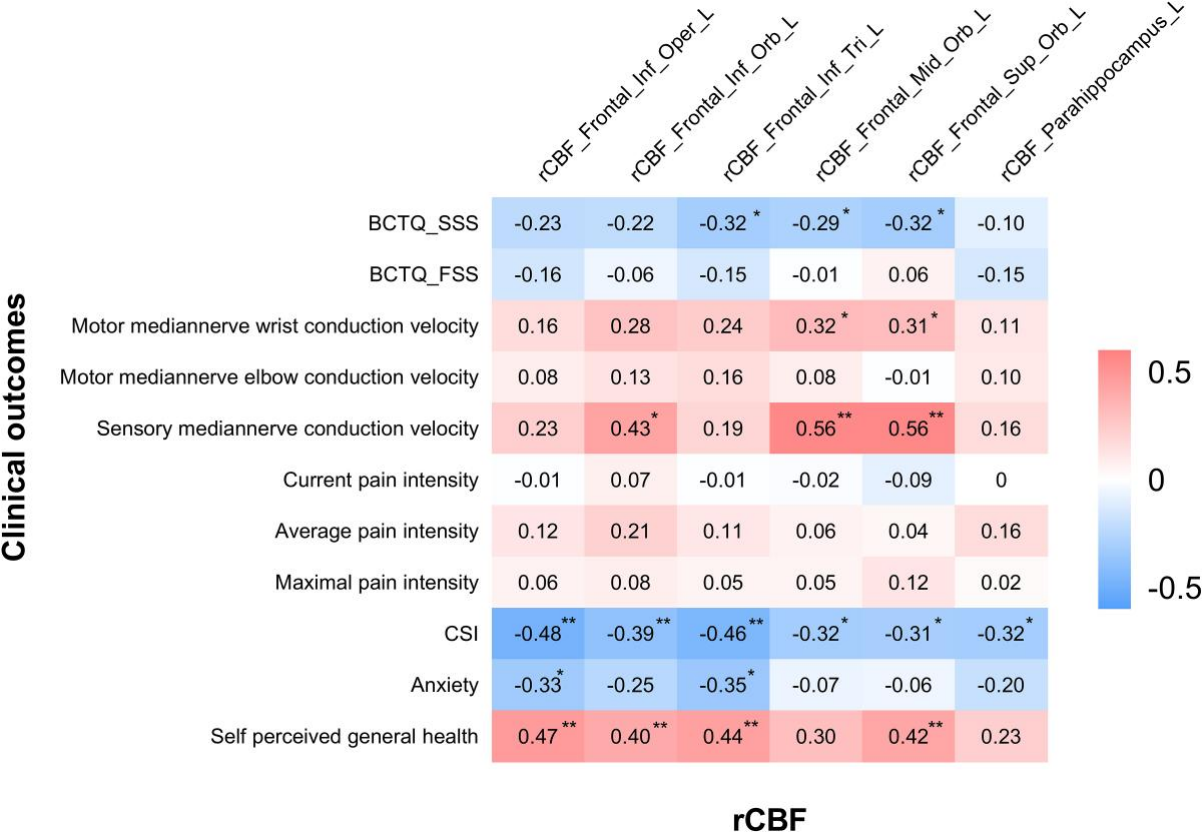
**Correlations between rCBF and clinical outcomes in CTS patients.** Correlations between rCBF and the symptom severity scale score of the BCTQ, nerve conduction study outcomes, pain ratings, central sensitization score according to the CSI, anxiety score and self-perceived general health score. Correlation analysis was conducted via Pearson or Spearman correlation, depending on the data distribution and normality, in patients with recurrent CTS (*n* = 49). Abbreviations: BCTQ_FSS, the functional status scale of Boston Carpal Tunnel Questionnaire; BCTQ_SSS, symptom severity scale of Boston Carpal Tunnel Questionnaire; CSI, central sensitization inventory; Frontal_Inf_Oper, inferior frontal gyrus, opercular part; Frontal_Inf_Orb, inferior frontal gyrus, orbital part; Frontal_Inf_Tri, inferior frontal gyrus, triangular part; Frontal_Mid_Orb, middle frontal gyrus, orbital part; Frontal_Sup_Orb, superior frontal gyrus, orbital part; parahippocampus, parahippocampal gyrus; rCBF, regional cerebral blood flow. *P < 0.05; **P < 0.01.

In terms of between-group differences in rCBF among different brain regions, significant negative correlations were found between rCBF in the left inferior frontal gyrus and clinical parameters such as CTS-specific symptom severity by BCTQ, central sensitization score and anxiety score. Similarly, rCBFs in the left middle and superior frontal gyrus were negatively associated with CTS-specific symptom severity and the central sensitization score. Furthermore, an inverse association was observed between rCBF in the left parahippocampus and the central sensitization score. Moreover, positive correlations were found between the rCBF in the left frontal gyrus and the motor nerve conduction velocities of the median nerve, the sensory nerve conduction velocity of the median nerve and self-perceived general health. No significant correlations were found between rCBF in the left thalamus and clinical outcomes in CTS patients. A summary of the correlational results between rCBF and clinical parameters can be found in Fig. 3.

**Figure 4:**
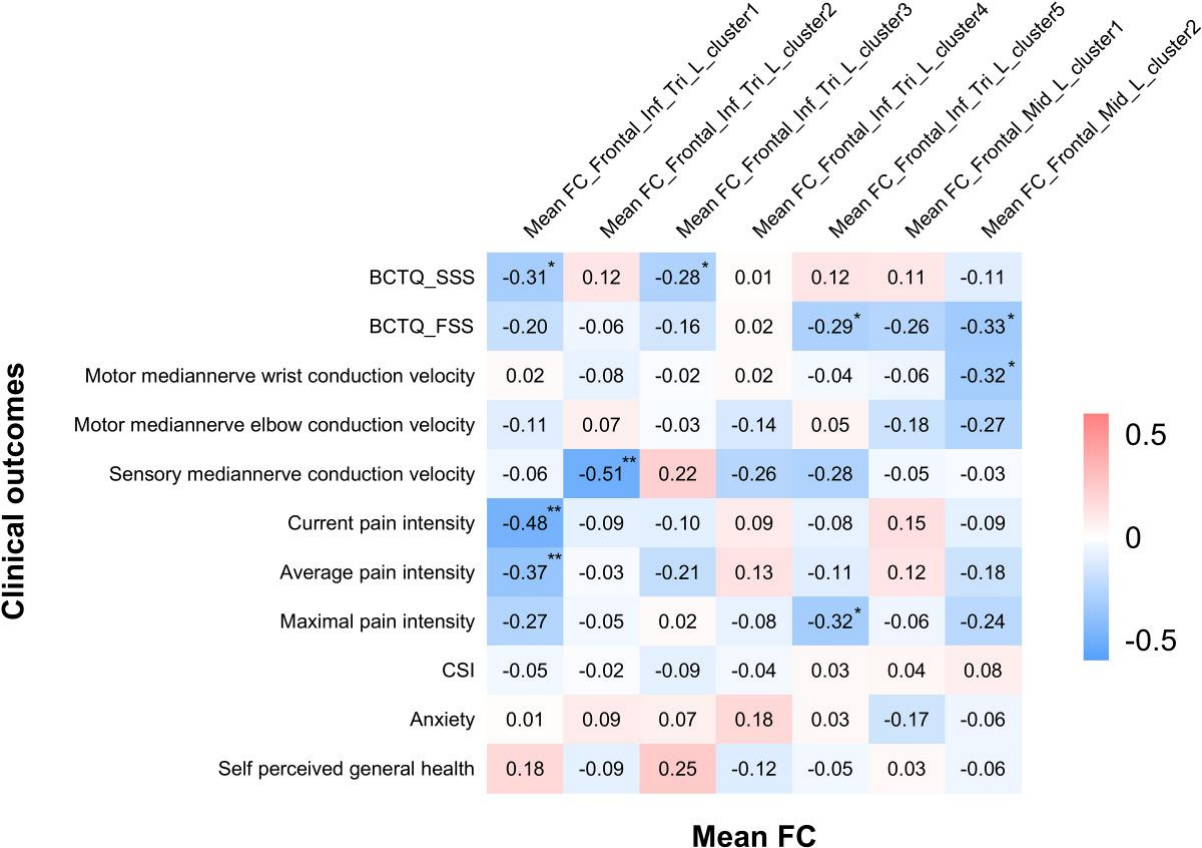
**Correlations between FC and clinical outcomes in CTS patients.** Correlations between the mean FC value and the symptom severity scale score of the BCTQ, nerve conduction study outcomes, pain ratings, central sensitization score according to the CSI, anxiety score and self-perceived general health score. Correlation analysis was conducted via Pearson or Spearman correlation, depending on the data distribution and normality, in patients with recurrent CTS (*n* = 49). Abbreviations: BCTQ_FSS, the functional status scale of Boston Carpal Tunnel Questionnaire; BCTQ_SSS, symptom severity scale of Boston Carpal Tunnel Questionnaire; CSI, central sensitization inventory; FC, functional connectivity; Frontal_Inf_Tri, triangular part of inferior frontal gyrus; Frontal_Mid, middle frontal gyrus. *P < 0.05; **P < 0.01.

With respect to the associations between mean FC and clinical parameters, inverse correlations were found between left inferior frontal gyrus to whole-brain FC and CTS-specific symptom severity, sensory nerve conduction velocity of the median nerve, current pain intensity, average pain intensity and worse pain intensity. Similarly, the mean left middle frontal gyrus to whole-brain FC was negatively associated with CTS-related function according to the BCTQ and motor nerve conduction velocities of the median nerve. The correlational results between the mean FC and CTS-related clinical outcomes can be found in Fig. 4.

## Discussion

To the best of our knowledge, this work is the first study to investigate the cortical mechanisms related to chronic neuropathic pain secondary to recurrent median nerve entrapment post carpal tunnel release surgeries via multimodal neuroimaging. Our main findings are as follows: (i) significantly decreased global CBF and rCBF in several brain regions, including the left (contralateral) inferior frontal gyrus, left middle frontal gyrus and left superior frontal gyrus, as well as the left parahippocampus and thalamus, were found among those with neuropathic pain post recurring CTS, compared with the healthy controls. An aberrant relCBF map was observed in the recurrent CTS group, involving brain areas such as the contralateral inferior and middle frontal gyrus and insula. (ii) Increased seed-to-voxel FC (the left inferior frontal gyrus and left middle frontal gyrus to the whole brain) was seen in the recurring CTS group in comparison to the controls. (iii) Significant negative associations were discovered between CBF alterations and clinical outcomes, including central sensitization score, anxiety, depression and CTS-specific symptom severity, whereas positive correlations were noted between CBF and NCS outcomes and self-perceived general health in the recurring CTS group. The seed-based FC in patients with CTS was inversely correlated with CTS symptom severity, NCS outcomes, functional status and pain intensity. The findings of the present study suggest the involvement of the prefrontal cortex, insular cortex and limbic system in the centrally modulating mechanisms involved in chronic neuropathic pain secondary to recurring CTS. The altered cerebral blood flow and functional connectivity patterns revealed by this study support the importance of recognizing the contributions of the central nervous system to recurrent painful median neuropathy.

Despite being a commonly performed surgical procedure, carpal tunnel release may sometimes result in suboptimal outcomes or lasting symptoms.^36^ The potential causes of surgical failure are multifactorial and complex, which may be divided into several aspects, including incomplete release of the transverse carpal ligament, perineural fibrosis, scar tissue formation, postoperative complications, unsatisfactory rehabilitation, psychosocial factors and central sensitization, among others.^36^ In the present study, recurrent CTS after the surgical procedure was confirmed by an experienced physician in the neurophysiological lab, taking into account three main dimensions, namely, clinical (prior history of carpal tunnel release, a symptom-free/relieving interval and reappearance of typical CTS symptoms and/or positive physical exams such as Tinel’s sign or Phalen’s test), electrophysiological (nerve conduction studies) and optional ultrasound imaging. Despite the lack of comparison results of NCV studies in patients before and after release surgery, there should have been a symptom-free interval after the release procedure in the study. As such, the major aim of this study was to investigate the potential central mediating mechanism of persistent neuropathic pain secondary to recurring CTS.

As one of the key findings in our study, an altered relCBF map was demonstrated in the contralateral PFC among participants with painful recurring median nerve entrapment. The role of the PFC in chronic pain has been revealed by compelling neuroimaging evidence,^37-43^ which indicates the cognitive and affective components of pain-related brain neural networks. The inferior frontal gyrus, which is part of the PFC, is a critical region involved not only in linguistics and motor control but also in cognitive and emotional processing.^40,43-45^ In our study, significantly decreased rCBF was observed in recurrent painful CTS patients in multiple regions encompassing the contralateral inferior frontal gyrus and the orbital part of the left superior and middle frontal gyrus in contrast to HCs. We also found markedly increased left inferior frontal gyrus to whole-brain FC in those with recurring CTS, extending connectivity with the bilateral superior medial frontal gyrus, left angular gyrus, left middle frontal gyrus and left inferior and middle temporal gyrus. In the correlational analysis, inverse associations were revealed between rCBF in the left inferior frontal gyrus and CTS-specific symptom severity, sensitization score and anxiety score. There was a positive correlation between rCBF in the left inferior frontal gyrus and self-perceived general health. In addition, negative associations were observed between the mean FC of the left inferior frontal gyrus and CTS-related symptom severity as well as self-reported pain intensity. As revealed by previous neuroimaging studies on the primary CTS population, abnormal primary somatosensory cortex (S1) neuroplasticity has been demonstrated in terms of structural morphology as well as functional connectivity between S1 and whole-brain regions.^13-15^ In contrast, our study provides additional insights into the modulating effect of the PFC on recurrent painful CTS after release surgery.

Another important finding of this study is that significantly reduced rCBF was demonstrated in the contralateral parahippocampal gyrus and thalamus among those with recurring CTS compared with HCs. As is known, disturbances in the limbic system, including the hippocampus, thalamus, cingulate gyrus and amygdala, have also been found in persistent pain disorders,^46-51^ which is consistent with our rCBF results. In addition to decreased CBF in the limbic system, we also found that the rCBF in the contralateral parahippocampal gyrus was inversely associated with the total CSI score. In terms of brain connectivity, significantly elevated seed-to-voxel FC was noted in the left middle frontal gyrus to the whole brain, including regions of the bilateral calcarine fissure and surrounding cortex and the bilateral middle cingulate and paracingulate gyri, in those with recurrent painful CTS. However, no significant differences were found between the two groups in terms of the seed-based FC with ACC and insula as the seed regions. It is suggested that the limbic system, especially the thalamus and parahippocampal gyrus, may also play a role in the centrally mediating chronic pain secondary to recurring CTS. This finding is partially in line with previous findings concerning the cortical responses of the primary CTS population in response to acupuncture stimuli, where the hypothalamic response positively correlated with maladaptive cortical plasticity in primary CTS.^52^

The insular cortex is a cortical hub involved in a series of crucial functions, such as somatosensory processing, autonomic control, self-awareness, emotion and behaviour, with multiple and complicated linkages to other brain regions.^53-55^ Cortical changes in the insula have been reported in individuals with psychiatric disorders and chronic pain.^55-60^ In the present study, we found a significantly altered relCBF map in the contralateral insular cortex in recurrent CTS patients. However, no significant difference was found in the rCBF of the left insula between recurring CTS patients and HCs, nor was it found in the seed-based FC between groups with the insula as the seed. Further investigations are needed to explore the relationship between the insular cortex and chronic painful neuropathy.

Notably, abnormal brain patterns caused by systemic peripheral nerve injury, such as painful diabetic polyneuropathy and small fibre neuropathy, have also been reported, revealing the important nodal role of the insula and limbic system in pain processing, which echoes the results of the present study.^61-64^ In systemic peripheral neuropathy, specific structural, functional and metabolic changes in S1 have been demonstrated. Similarly, S1-related neuroplasticity was noted in primary CTS,^12-14^ whereas among the recurring CTS population in this study, a distinct brain alteration pattern was disclosed in the PFC, insula, parahippocampal gyrus and thalamus. Future studies are warranted to look into the differentiation of neuropathy secondary to primary focal nerve injury and systemic disorders, as well as recurring neuropathy.

In addition, the coexistence of negative emotions such as anxiety and depression is not uncommon in people suffering from chronic pain disorders.^42,65-67^ In the present study, participants with recurrent painful CTS had significantly higher anxiety and depression scores than HCs did. Moreover, the rCBF in the contralateral inferior frontal gyrus was inversely associated with the anxiety score in the CTS group. On the one hand, individuals with ongoing pain symptoms are more vulnerable to developing anxiety or depression. On the other hand, the comorbidity of anxiety and/or depression may have an adverse effect on pain perception and experience, which makes the pain-coping approach more complicated and challenging.^65,67^ The elevated anxiety status found in recurring CTS patients is also in line with our findings, indicating the potential role of several brain regions involved in emotional, affective and cognitive control, such as the PFC and the limbic system.

There are several strengths in the present study. First of all, this study focused on a specific subgroup of recurring painful CTS patients post carpal release surgery, which is a critically challenging issue for clinicians. To the best of our knowledge, the present work is the first to investigate the potential central mechanisms underlying recurrent median neuropathy in a sample of patients with moderate to severe recurrent CTS suffering from chronic neuropathic pain. Second, our study revealed altered neuronal activity in brain regions in the recurring CTS population beyond the somatosensory cortex, which was observed in non-recurrent CTS individuals. Maladaptive plasticity changes were observed in brain regions, including the PFC, insular cortex, parahippocampal gyrus and thalamus, indicating the contributions of cognitive and affective processing in addition to somatosensory control. The abovementioned findings add new insights into refractory neuropathic pain secondary to recurring median nerve neuropathy. In addition, the comorbidity of anxiety and depression was also noted in chronic neuropathic pain in the present study, which echoed the cortical alterations in our neuroimaging results.

Several limitations exist as well. First, the sample size in the study is not large, although the *post hoc* power analysis showed good statistical power of 95%, which may ensure the validity of our findings. Moreover, only female patients with CTS were eligible for final inclusion in this consecutive cohort, which limits the generalizability of the results. However, these findings may indicate the phenomenon that women are more susceptible to experiencing recurrent compression neuropathy. Moreover, a non-recurrent CTS group or pain-free CTS group was lacking in this study, and further study would be recommended to consider adding positive comparators to elaborate on the specific brain network related to persistent neuropathic pain. The lack of a comparison of NVS results before and after carpal release surgery may limit accurate and objective assessments of surgical outcomes in the present study. Quantitative sensory testing was not conducted in our study, which placed a limit on the objective evaluation of central sensitization in recurrent CTS patients. In terms of the study design, the cross-sectional study design fails to keep an observation of the dynamic changes of neuroplasticity over time. In light of this, future longitudinal trials are warranted to investigate the alterations in brain plasticity after interventions for chronic neuropathic pain after recurring median nerve entrapment.

## Conclusion

This study demonstrated that recurrent painful CTS patients with chronic neuropathic pain presented altered CBF coupled with maladaptive brain connectivity in brain regions involving sensory, cognitive and affective processing, such as the PFC and limbic system. Our findings may provide important insights into potential mechanisms underlying persistent neuropathic pain after recurrent neuropathy, which may facilitate alternative, centrally targeted management strategies.

## Supplementary Material

fcaf375_Supplementary_Data

## Data Availability

All data associated with this study are presented in the manuscript or the Supplementary material. The raw datasets are available from the corresponding author on reasonable request. All MRI image processing and analyses were performed on the MATLAB platform (MATLAB version 2021b, MathWorks Inc., Natick, MA, USA; https://www.mathworks.com). The Data Processing and Analysis of Brain Imaging Toolbox (https://rfmri.org/DPABI) was used to process resting-state fMRI data. The pCASL perfusion images were preprocessed via a modified processing pipeline, ASLtbx27 (https://www.cfn.upenn.edu/zewang/ASLtbx.php), which is based on the Statistical Parametric Mapping 12 (SPM12, Functional Imaging Laboratory, Oxford, UK; https://www.fil.ion.ucl.ac.uk/spm/software/spm12). The SPSS 27 (SPSS Inc., Chicago, IL, USA; https://spss.en.softonic.com) was used for statistical analysis.

## References

[1] Feng B, Chen K, Zhu X, et al Prevalence and risk factors of self-reported wrist and hand symptoms and clinically confirmed carpal tunnel syndrome among office workers in China: A cross-sectional study. BMC Public Health. 2021;21(1):52–57. 33407293 10.1186/s12889-020-10137-1 PMC7789363

[2] Wipperman J, Penny ML. Carpal tunnel syndrome: Rapid evidence review. Am Fam Physician. 2024;110(1):52–57. 39028782

[3] Padua L, Coraci D, Erra C, et al Carpal tunnel syndrome: Clinical features, diagnosis, and management. Lancet Neurol. 2016;15(12):1273–1284. 27751557 10.1016/S1474-4422(16)30231-9

[4] Shapiro LM, Kamal RN. American Academy of Orthopaedic Surgeons/ASSH clinical practice guideline summary management of carpal tunnel syndrome. J Am Acad Orthop Surg. 2025;33(7):e356–e366. 39637428 10.5435/JAAOS-D-24-01179 PMC11928260

[5] Sydora B, Whelan L, Abelseth B, et al Identification of presurgical risk factors for the development of chronic postsurgical pain in adults: A comprehensive umbrella review. J Pain Res. 2024;17:2511–2530. 39100136 10.2147/JPR.S466731 PMC11297490

[6] Westenberg RF, DiGiovanni PL, Schep NWL, Eberlin KR, Chen NC, Coert JH. Does revision carpal tunnel release result in long-term outcomes equivalent to single carpal tunnel release? A matched case-control analysis. Plast Reconstr Surg. 2024;153(4):746e–757e. 10.1097/PRS.0000000000010682 37189245

[7] Ferrin PC, Sather BK, Krakauer K, Schweitzer TP, Lipira AB, Sood RF. Revision carpal tunnel release following endoscopic compared with open decompression. JAMA Netw Open. 2024;7(1):e2352660. 38214927 10.1001/jamanetworkopen.2023.52660 PMC10787312

[8] Chan E, Billard K, Sims L, Yang C, Sauder D. Does addition of a longer acting local anesthetic improve postoperative pain after carpal tunnel release? A randomized controlled trial. J Hand Surg Am. 2024;49(10): 1000–1006. 38958611 10.1016/j.jhsa.2024.05.009

[9] Fernández-de-Las-Peñas C, Plaza-Manzano G. Carpal tunnel syndrome: Just a peripheral neuropathy? Pain Manag. 2018;8(3):209–216. 29869575 10.2217/pmt-2017-0063

[10] Osborne NR, Anastakis DJ, Kim JA, et al Carpal tunnel surgery dampens thalamocortical and normalizes corticocortical functional connectivity. Brain Commun. 2022;4(5):fcac237. 36246046 10.1093/braincomms/fcac237 PMC9556937

[11] Sobeeh MG, Ghozy S, Elshazli RM, Landry M. Pain mechanisms in carpal tunnel syndrome: A systematic review and meta-analysis of quantitative sensory testing outcomes. Pain. 2022;163(10):e1054–e1094. 35050958 10.1097/j.pain.0000000000002566

[12] Lu YC, Zhang H, Zheng MX, et al Local and extensive neuroplasticity in carpal tunnel syndrome: A resting-state fMRI study. Neurorehabil Neural Repair. 2017;31(10-11):898–909. 28845734 10.1177/1545968317723749

[13] Maeda Y, Kettner N, Kim J, et al Primary somatosensory/motor cortical thickness distinguishes paresthesia-dominant from pain-dominant carpal tunnel syndrome. Pain. 2016;157(5):1085–1093. 26761384 10.1097/j.pain.0000000000000486

[14] Maeda Y, Kim H, Kettner N, et al Rewiring the primary somatosensory cortex in carpal tunnel syndrome with acupuncture. Brain. 2017;140(4):914–927. 28334999 10.1093/brain/awx015 PMC5837382

[15] Li YL, Wu JJ, Ma J, et al Brain structural changes in carpal tunnel syndrome patients: From the perspectives of structural connectivity and structural covariance network. Neurosurgery. 2021;89(6):978–986. 34634107 10.1093/neuros/nyab335

[16] Sobeeh MG, Benmelouka A, Metwally E, et al Altered brain function and structure in carpal tunnel syndrome: A systematic review and meta-analysis of structural and functional brain imaging. Brain Struct Funct. 2024;229(2):257–272. 38165482 10.1007/s00429-023-02737-5

[17] Xue X, Wu JJ, Hua XY, et al Structural white matter alterations in carpal tunnel syndrome: A modified TBSS study. Brain Res. 2021;1767:147558. 34116054 10.1016/j.brainres.2021.147558

[18] Xing XX, Hua XY, Zheng MX, et al Abnormal brain connectivity in carpal tunnel syndrome assessed by graph theory. J Pain Res. 2021;14:693–701. 33732015 10.2147/JPR.S289165 PMC7959208

[19] Chen X, Lu B, Yan CG. Reproducibility of R-fMRI metrics on the impact of different strategies for multiple comparison correction and sample sizes. Hum Brain Mapp. 2018;39(1):300–318. 29024299 10.1002/hbm.23843 PMC6866539

[20] Chiarotto A, Maxwell LJ, Ostelo RW, Boers M, Tugwell P, Terwee CB. Measurement properties of visual analogue scale, numeric rating scale, and pain severity subscale of the brief pain inventory in patients with low back pain: A systematic review. J Pain. 2019;20(3):245–263. 30099210 10.1016/j.jpain.2018.07.009

[21] Feng B, Hu X, Lu WW, Wang Y, Ip WY. Cultural validation of the Chinese central sensitization inventory in patients with chronic pain and its predictive ability of comorbid central sensitivity syndromes. J Pain Res. 2022;15:467–477. 35210847 10.2147/JPR.S348842 PMC8857991

[22] Leung CM, Wing YK, Kwong PK, Lo A, Shum K. Validation of the Chinese-Cantonese version of the hospital anxiety and depression scale and comparison with the Hamilton rating scale of depression. Acta Psychiatr Scand. 1999;100(6):456–61. 10626925 10.1111/j.1600-0447.1999.tb10897.x

[23] Zhu J, Yan XX, Liu CC, et al Comparing EQ-5D-3L and EQ-5D-5L performance in common cancers: Suggestions for instrument choosing. Qual Life Res. 2021;30(3):841–854. 32930993 10.1007/s11136-020-02636-w

[24] Deng X, Chau LP, Chiu SY, Leung KP, Hu Y, Ip WY. Screening of axonal degeneration in carpal tunnel syndrome using ultrasonography and nerve conduction studies. J Vis Exp. 2019; doi:10.3791/58681 30688308

[25] Feng B, Gong C, You L, et al Central sensitization in patients with chronic pain secondary to carpal tunnel syndrome and determinants. J Pain Res. 2023;16:4353–4366. 38145037 10.2147/JPR.S441786 PMC10748611

[26] Bland JD . A neurophysiological grading scale for carpal tunnel syndrome. Muscle Nerve. 2000;23(8):1280–3. 10918269 10.1002/1097-4598(200008)23:8<1280::aid-mus20>3.0.co;2-y

[27] McCallum LM, Damms NA, Sarrigiannis PG, Zis P. Anxiety and depression in patients with suspected carpal tunnel syndrome—A case controlled study. Brain Behav. 2019;9(7):e01342. 31210031 10.1002/brb3.1342 PMC6625534

[28] Wang Z . Improving cerebral blood flow quantification for arterial spin labeled perfusion MRI by removing residual motion artifacts and global signal fluctuations. Magn Reson Imaging. 2012;30(10):1409–15. 22789842 10.1016/j.mri.2012.05.004 PMC3482282

[29] Tzourio-Mazoyer N, Landeau B, Papathanassiou D, et al Automated anatomical labeling of activations in SPM using a macroscopic anatomical parcellation of the MNI MRI single-subject brain. Neuroimage. 2002;15(1):273–89. 11771995 10.1006/nimg.2001.0978

[30] Yan CG, Wang XD, Zuo XN, Zang YF. DPABI: Data processing & analysis for (resting-state) brain imaging. Neuroinformatics. 2016;14(3):339–51. 27075850 10.1007/s12021-016-9299-4

[31] Lee M, Manders TR, Eberle SE, et al Activation of corticostriatal circuitry relieves chronic neuropathic pain. J Neurosci. 2015;35(13):5247–59. 25834050 10.1523/JNEUROSCI.3494-14.2015 PMC4380998

[32] Metz AE, Yau HJ, Centeno MV, Apkarian AV, Martina M. Morphological and functional reorganization of rat medial prefrontal cortex in neuropathic pain. Proc Natl Acad Sci U S A. 2009;106(7):2423–8. 19171885 10.1073/pnas.0809897106 PMC2650172

[33] Shiers S, Price TJ. Molecular, circuit, and anatomical changes in the prefrontal cortex in chronic pain. Pain. 2020;161(8):1726–1729. 32701833 10.1097/j.pain.0000000000001897 PMC7575617

[34] Tan LL, Kuner R. Neocortical circuits in pain and pain relief. Nat Rev Neurosci. 2021;22(8):458–471. 34127843 10.1038/s41583-021-00468-2

[35] Liu P, Uh J, Devous MD, Adinoff B, Lu H. Comparison of relative cerebral blood flow maps using pseudo-continuous arterial spin labeling and single photon emission computed tomography. NMR Biomed. 2012;25(5):779–786. 22139764 10.1002/nbm.1792 PMC3298573

[36] Decramer A, Heras-Palou C, Van Nuffel M, Lattré T, Degreef I. Management of failed carpal tunnel decompression. EFORT Open Rev. 2025;10(6):352–360. 40459165 10.1530/EOR-2025-0058 PMC12139599

[37] Kang D, Hesam-Shariati N, McAuley JH, et al Disruption to normal excitatory and inhibitory function within the medial prefrontal cortex in people with chronic pain. Eur J Pain. 2021;25(10):2242–2256. 34242465 10.1002/ejp.1838

[38] Ashar YK, Gordon A, Schubiner H, et al Effect of pain reprocessing therapy vs placebo and usual care for patients with chronic back pain: A randomized clinical trial. JAMA Psychiatry. 2022;79(1):13–23. 34586357 10.1001/jamapsychiatry.2021.2669 PMC8482298

[39] Naylor B, Hesam-Shariati N, McAuley JH, et al Reduced glutamate in the medial prefrontal Cortex is associated with emotional and cognitive dysregulation in people with chronic pain. Front Neurol. 2019;10:1110. 31849800 10.3389/fneur.2019.01110 PMC6903775

[40] Zhou J, Wang Y, Luo X, et al Revisiting the effects of rTMS over the dorsolateral prefrontal cortex on pain: An updated systematic review and meta-analysis. Brain Stimul. 2024;17(4):928–937. 39089648 10.1016/j.brs.2024.07.011

[41] Ma J, Hua XY, Zheng MX, et al Surface-based map plasticity of brain regions related to sensory motor and pain information processing after osteonecrosis of the femoral head. Neural Regen Res. 2022;17(4):806–811. 34472479 10.4103/1673-5374.322471 PMC8530129

[42] Du Y, Xu CL, Yu J, et al Hmgb1 in the mPFC governs comorbid anxiety in neuropathic pain. J Headache Pain. 2022;23(1):102. 35974316 10.1186/s10194-022-01475-z PMC9382735

[43] Vats D, Bhatia R, Fatima S, et al Repetitive transcranial magnetic stimulation of the dorsolateral prefrontal Cortex for phantom limb pain. Pain Physician. 2024;27(5):E589–E595. 39087968

[44] Briggs RG, Chakraborty AR, Anderson CD, et al Anatomy and white matter connections of the inferior frontal gyrus. Clin Anat. 2019;32(4):546–556. 30719769 10.1002/ca.23349

[45] Li Y, Seger C, Chen Q, Mo L. Left Inferior frontal gyrus integrates multisensory information in category learning. Cereb Cortex. 2020;30(8):4410–4423. 32133488 10.1093/cercor/bhaa029

[46] Verriotis M, Sorger C, Peters J, et al Amygdalar functional connectivity differences associated with reduced pain intensity in pediatric peripheral neuropathic pain. Front Pain Res (Lausanne). 2022;3:918766. 35692562 10.3389/fpain.2022.918766 PMC9184677

[47] Meeker TJ, Schmid AC, Keaser ML, et al Tonic pain alters functional connectivity of the descending pain modulatory network involving amygdala, periaqueductal gray, parabrachial nucleus and anterior cingulate cortex. Neuroimage. 2022;256:119278. 35523367 10.1016/j.neuroimage.2022.119278 PMC9250649

[48] Wang JW, Huang ZQ, Lu YJ, Sha K, Li WM, Zhao JM. Cerebral gray matter volume changes in patients with neuropathic pain from total brachial Plexus injury. Eur Neurol. 2023;86(1):45–54. 35901777 10.1159/000525527

[49] Mosch B, Hagena V, Herpertz S, Ruttorf M, Diers M. Neural correlates of control over pain in fibromyalgia patients. Neuroimage Clin. 2023;37:103355. 36848728 10.1016/j.nicl.2023.103355 PMC9982683

[50] Ninneman JV, Gretzon NP, Stegner AJ, et al Pain, but not physical activity, is associated with gray matter volume differences in Gulf War veterans with chronic pain. J Neurosci. 2022;42(28):5605–5616. 35697521 10.1523/JNEUROSCI.2394-21.2022 PMC9295831

[51] Sloan G, Teh K, Caunt S, Wilkinson I, Selvarajah D, Tesfaye S. Increased thalamocortical functional connectivity on discontinuation of treatment in painful diabetic peripheral neuropathy. Diabetes. 2024;73(9): 1486–1494. 38905144 10.2337/db23-0931

[52] Napadow V, Kettner N, Liu J, et al Hypothalamus and amygdala response to acupuncture stimuli in carpal tunnel syndrome. Pain. 2007;130(3):254–266. 17240066 10.1016/j.pain.2006.12.003 PMC1997288

[53] Benarroch EE . Insular cortex: Functional complexity and clinical correlations. Neurology. 2019;93(21):932–938. 31645470 10.1212/WNL.0000000000008525

[54] Holtmann O, Franz M, Mönig C, et al Lateralized deficits in arousal processing after insula lesions: Behavioral and autonomic evidence. Cortex. Mar. 2022;148:168–179. 10.1016/j.cortex.2021.12.013 35180480

[55] Mammadkhanli O, Kehaya S, Solak S, Yağmurlu K. Insular cortex involvement in migraine patients with chronic pain: A volumetric radiological and clinical study. J Clin Neurosci. 2024;123:157–161. 38579522 10.1016/j.jocn.2024.03.034

[56] Nagasaka K, Otsuru N, Sato R, et al Cortical signature related to psychometric properties of pain vigilance in healthy individuals: A voxel-based morphometric study. Neurosci Lett. 2022;772:136445. 35007688 10.1016/j.neulet.2022.136445

[57] Lee GJ, Kim YJ, Shim SW, Lee K, Oh SB. Anterior insular-nucleus accumbens pathway controls refeeding-induced analgesia under chronic inflammatory pain condition. Neuroscience. 2022;495:58–73. 35643248 10.1016/j.neuroscience.2022.05.025

[58] Horing B, Büchel C. The human insula processes both modality-independent and pain-selective learning signals. PLoS Biol. 2022;20(5):e3001540. 35522696 10.1371/journal.pbio.3001540 PMC9116652

[59] Mammadkhanli O, Niftaliyev S, Simsek O. Involvement of the cingulate cortex and insula in patients with trigeminal neuralgia: A clinical and volumetric study. Clin Neurol Neurosurg. 2024;243:108394. 38908321 10.1016/j.clineuro.2024.108394

[60] Tretyak V, Kirsch DE, Le V, Fromme K, Strakowski SM, Lippard ETC. Coping drinking motives, neural functional coupling during emotion processing, and alcohol use in young adults with bipolar disorder. Alcohol Clin Exp Res. 2022;46(8):1482–1496. 35702929 10.1111/acer.14885 PMC9478569

[61] Teh K, Wilkinson ID, Heiberg-Gibbons F, et al Somatosensory network functional connectivity differentiates clinical pain phenotypes in diabetic neuropathy. Diabetologia. 2021;64(6):1412–1421. 33768284 10.1007/s00125-021-05416-4 PMC8099810

[62] Sloan G, Anton A, Caunt S, Wilkinson I, Selvarajah D, Tesfaye S. Higher sensory cortical energy metabolism in painful diabetic neuropathy: Evidence from a cerebral magnetic resonance spectroscopy study. Diabetes. 2023;72(7):1028–1034. 37058464 10.2337/db23-0051

[63] Chao CC, Hsieh PC, Janice Lin CH, Huang SL, Hsieh ST, Chiang MC. Limbic connectivity underlies pain treatment response in small-fiber neuropathy. Ann Neurol. 2023;93(4):655–667. 36511844 10.1002/ana.26577

[64] Selvarajah D, Sloan G, Teh K, et al Structural brain alterations in key somatosensory and nociceptive regions in diabetic peripheral neuropathy. Diabetes Care. 2023;46(4):777–785. 36749934 10.2337/dc22-1123

[65] Qiu Y, Ma Y, Huang X. Bidirectional relationship between body pain and depressive symptoms: A pooled analysis of two national aging cohort studies. Front Psychiatry. 2022;13:881779. 35558432 10.3389/fpsyt.2022.881779 PMC9086823

[66] Maunder L, Pavlova M, Beveridge JK, Katz J, Salomons TV, Noel M. Sensitivity to pain traumatization and its relationship to the anxiety-pain connection in youth with chronic pain: Implications for treatment. Children (Basel). 2022;9(4):529. 35455573 10.3390/children9040529 PMC9032504

[67] Zhu X, Zhang C, Hu Y, et al Modulation of comorbid chronic neuropathic pain and anxiety-like behaviors by glutamatergic neurons in the vlPAG and the analgesic and anxiolytic effects of EA. eNeuro. 2024;11(8): ENEURO.0454-23.2024. 10.1523/ENEURO.0454-23.2024 PMC11360982 39084906

