# Supplementary material for: Altered patterns in the prefrontal cortex and limbic system in female patients with neuropathic pain secondary to recurrent median nerve entrapment post carpal tunnel release
Source: Brain Commun. 2025 Sep 26;7(6):fcaf375. doi: 10.1093/braincomms/fcaf375 (PMC12606578; doi:10.1093/braincomms/fcaf375)
Supplement: fcaf375_Supplementary_Data [file fcaf375_supplementary_data.docx]

**Supplements**

**Table S1 Global CBF between CTS patients and HCs**

|  | CTS | HC | *P* value |
| --- | --- | --- | --- |
| Mean global CBF (ml/100g/min,  mean (SD)) | 41.9 (7.23) | 45.9 (5.74) | 0.024^*^ |

Abbreviations: CBF: cerebral blood flow; CTS: carpal tunnel syndrome; HC: healthy control; SD: standard deviation.

Note: *represents p <0.05 for between-group comparisons.

**Table S2** **Brain regions of significantly different relative CBF between CTS and HC groups**

| Brain region | Cluster size | Peak MNI coordinates (mm) | | | Peak T value | *P* value |
| --- | --- | --- | --- | --- | --- | --- |
|  |  | X | Y | Z |  |  |
|  | 461 | -40 | 36 | 18 | 4.4133 | < 0.05 |
| Frontal_Inf_Tri_L |  |  |  |  |  |  |
| Frontal_Mid_L  Insula_L |  |  |  |  |  |  |

Abbreviations: CBF: cerebral blood flow; CTS: carpal tunnel syndrome; HC: healthy control; MNI: Montréal Neurological Institute; Frontal_Inf_Tri_L: left triangular part of inferior frontal gyrus; Frontal_Mid_L: left middle frontal gyrus; Insula_L: left insular cortex.

Note: Two sample T test was used to compare the difference in relative CBF between CTS and HC groups with age as the covariate and the multiple comparison was corrected by Gaussian Random Field method with voxel p <0.001, cluster p <0.05, two-tailed.

**Table S3 Brain regions showing significant** **Frontal_Inf_Tri_L: whole brain FC between CTS patients and** **HCs**

| Brain region | Cluster size | Peak MNI coordinates (mm) | | | Peak T value | P value |
| --- | --- | --- | --- | --- | --- | --- |
|  |  | X | Y | Z |  |  |
| Cluster 1 | 147 | -6 | 30 | 36 | 4.7529 | < 0.05 |
| Frontal_Sup_Medial_L |  |  |  |  |  |  |
| Frontal_Sup_Medial_R |  |  |  |  |  |  |
| Cluster 2 | 44 | -39 | -57 | 33 | 4.1741 | < 0.05 |
| Angular_L |  |  |  |  |  |  |
| Cluster 3 | 39 | -36 | 54 | 6 | 4.1335 | < 0.05 |
| Frontal_Mid_L |  |  |  |  |  |  |
| Cluster 4 | 73 | -33 | 21 | 39 | 4.9203 | < 0.05 |
| Frontal_Mid_L |  |  |  |  |  |  |
| Cluster 5 | 47 | -54 | -18 | -24 | 4.9236 | < 0.05 |
| Temporal_Inf_L |  |  |  |  |  |  |
| Temporal_Mid_L |  |  |  |  |  |  |

Abbreviations: Frontal_Inf_Tri_L: left triangular part of inferior frontal gyrus; FC: functional connectivity; CTS: carpal tunnel syndrome; HC: healthy control; MNI: Montréal Neurological Institute; Frontal_Sup_Medial_L: left superior frontal gyrus, medial; Frontal_Sup_Medial_R: right superior frontal gyrus, medial; Angular_L: left angular gyrus; Frontal_Mid_L: left middle frontal gyrus; Temporal_Inf_L: left inferior temporal gyrus; Temporal_Mid_L: left middle temporal gyrus.

Note: Two sample T test was used to compare the difference in Frontal_Inf_Tri_L: whole brain FC between CTS and HC groups with age as the covariate and the multiple comparison was corrected by Gaussian Random Field method with voxel p <0.001, cluster p <0.05, two-tailed.

**Table S4 Brain regions showing significant** **Frontal_Mid_L: whole brain FC between CTS patients and HCs**

| Brain region | Cluster size | Peak MNI coordinates (mm) | | | Peak T value | *P* value |
| --- | --- | --- | --- | --- | --- | --- |
|  |  | X | Y | Z |  |  |
| Cluster 1 | 66 | -12 | -63 | 15 | 3.9038 | < 0.05 |
| Calcarine_L |  |  |  |  |  |  |
| Calcarine_R |  |  |  |  |  |  |
| Cluster 2 | 60 | 3 | -33 | 33 | 4.4078 | < 0.05 |
| Cingulum_Mid_R |  |  |  |  |  |  |
| Cingulum_Mid_L |  |  |  |  |  |  |

Abbreviations: Frontal_Mid_L: left middle frontal gyrus; FC: functional connectivity; CTS: carpal tunnel syndrome; HC: healthy control; MNI: Montréal Neurological Institute; Calcarine_L: left calcarine fissue and surrounding cortex; Calcarine_R: right calcarine fissue and surrounding cortex; Cingulate_Mid_R: right middle cingulate and paracingulate gyri; Cingulate_Mid_L: left middle cingulate and paracingulate gyri.

Note: Two sample T test was used to compare the difference in Frontal_Mid_L: whole brain FC between CTS and HC groups with age as the covariate and the multiple comparison was corrected by Gaussian Random Field method with voxel p <0.001, cluster p <0.05, two-tailed.

**Table S5 Brain regions showing significant correlation between relative CBF and central sensitization by CSI in CTS patients**

| Brain region | Cluster size | Peak MNI coordinates (mm) | | | Peak intensity | *P* value |
| --- | --- | --- | --- | --- | --- | --- |
|  |  | X | Y | Z |  |  |
| Cluster 1 | 422 | 48 | 18 | 24 | -0.56958 | < 0.05 |
| Frontal_Inf_Tri_R |  |  |  |  |  |  |
| Frontal_Mid_R |  |  |  |  |  |  |
| Frontal_Inf_Oper_R |  |  |  |  |  |  |

Abbreviations: CBF: cerebral blood flow; CSI: central sensitization inventory; CTS: carpal tunnel syndrome; MNI: Montréal Neurological Institute; Frontal_Inf_Tri_R: right triangular part of inferior frontal gyrus; Frontal_Mid_R: right middle frontal gyrus; Frontal_Inf_Oper_R: right opercular part of inferior frontal gyrus.

Note: The partial correlational analysis was used to investigate the relationship between rCBFmap in CTS patients and the CSI scoring with age as the covariate, and the multiple comparison was corrected by Gaussian Random Field method with voxel p <0.001, cluster p <0.05, two-tailed.

**Table S6 Brain regions showing significant correlation between relative CBF and anxiety status by HADS in CTS patients**

| Brain region | Cluster size | Peak MNI coordinates (mm) | | | Peak intensity | *P* value |
| --- | --- | --- | --- | --- | --- | --- |
|  |  | X | Y | Z |  |  |
| Cluster 1 | 564 | 52 | 16 | 20 | -0.65781 | < 0.05 |
| Frontal_Inf_Oper_R |  |  |  |  |  |  |
| Frontal_Inf_Tri_R |  |  |  |  |  |  |
| Frontal_Mid_R |  |  |  |  |  |  |

Abbreviations: CBF: cerebral blood flow; HADS: Hospital Anxiety and Depression Scale; CTS: carpal tunnel syndrome; MNI: Montréal Neurological Institute; Frontal_Inf_Oper_R: right opercular part of inferior frontal gyrus; Frontal_Inf_Tri_R: right triangular part of inferior frontal gyrus; Frontal_Mid_R: right middle frontal gyrus.

Note: The partial correlational analysis was used to investigate the relationship between rCBFmap in CTS patients and the anxiety score with age as the covariate, and the multiple comparison was corrected by Gaussian Random Field method with voxel p <0.001, cluster p <0.05, two-tailed.

**Table S7 Brain regions showing significant correlation between relative CBF and depression status by HADS in CTS patients**

| Brain region | Cluster size | Peak MNI coordinates (mm) | | | Peak intensity | *P* value |
| --- | --- | --- | --- | --- | --- | --- |
|  |  | X | Y |  |  |  |
| Cluster 1 | 538 | 60 | -16 | 28 | -0.56987 | < 0.05 |
| SupraMarginal_R |  |  |  |  |  |  |
| Postcentral_R |  |  |  |  |  |  |
| Cluster 2 | 497 | 48 | 36 | 28 | -0.69178 | < 0.05 |
| Frontal_Inf_Tri_R |  |  |  |  |  |  |
| Frontal_Mid_R |  |  |  |  |  |  |

Abbreviations: CBF: cerebral blood flow; HADS: Hospital Anxiety and Depression Scale; CTS: carpal tunnel syndrome; MNI: Montréal Neurological Institute; SupraMarginal_R: right supramarginal gyrus; Postcentral_R: right postcentral gyrus; Frontal_Inf_Tri_R: right triangular part of inferior frontal gyrus; Frontal_Mid_R: right middle frontal gyrus.

Note: The partial correlational analysis was used to investigate the relationship between rCBFmap in CTS patients and the depression score with age as the covariate, and the multiple comparison was corrected by Gaussian Random Field method with voxel p <0.001, cluster p <0.05, two-tailed.

**Table S8 Brain regions showing significant correlation between relative CBF and self-perceived general health in CTS patients**

| Brain region | luster size | Peak MNI coordinates (mm) | | | Peak intensity | *P* value |
| --- | --- | --- | --- | --- | --- | --- |
|  |  | X | Y | Z |  |  |
| Cluster 1 | 1756 | 38 | 44 | 18 | 0.69195 | < 0.05 |
| Frontal_Mid_R |  |  |  |  |  |  |
| Frontal_Inf_Tri_R |  |  |  |  |  |  |
| Frontal_Inf_Oper_R |  |  |  |  |  |  |
| Cluster 2 | 914 | 54 | -30 | 30 | 0.65428 | < 0.05 |
| SupraMarginal_R |  |  |  |  |  |  |
| Temporal_Sup_R |  |  |  |  |  |  |
| Temporal_Mid_R |  |  |  |  |  |  |
| Parietal_Inf_R |  |  |  |  |  |  |

Abbreviations: CBF: cerebral blood flow; CTS: carpal tunnel syndrome; MNI: Montréal Neurological Institute; Frontal_Mid_R: right middle frontal gyrus; Frontal_Inf_Tri_R: right triangular part of inferior frontal gyrus; Frontal_Inf_Oper_R: right opercular part of inferior frontal gyrus; SupraMarginal_R: right supramarginal gyrus; Temporal_Sup_R: right superior temporal gyrus; Temporal_Mid_R: right middle temporal gyrus; Parietal_Inf_R: right inferior parietal, but supramarginal and angular gyri.

Note: The partial correlational analysis was used to investigate the relationship between rCBFmap in CTS patients and their self-perceived general health with age as the covariate, and the multiple comparison was corrected by Gaussian Random Field method with voxel p <0.001, cluster p <0.05, two-tailed.
